# Predictive analysis of dominant hand grip strength among young children aged 6–15 years using machine learning techniques: a decision tree and regression analysis

**DOI:** 10.3389/fped.2025.1569913

**Published:** 2025-03-19

**Authors:** Mastour Saeed Alshahrani, Resmi Ann Thomas, Paul Silvian Samuel, Venkata Nagaraj Kakaraparthi, Ravi Shankar Reddy, Snehil Dixit

**Affiliations:** ^1^ Department of Medical Rehabilitation Sciences, King Khalid University, Abha, Saudi Arabia; ^2^ Marketing and Retail Studies, The Business School, Centennial College, Toronto, ON, Canada

**Keywords:** hand grip strength, gender, dominant hand, decision tree, regression analysis

## Abstract

**Background:**

This study aimed to investigate and understand predictor variables and isolate the exact roles of anthropometric and demographic variables in the hand grip strength of young children.

**Material and methods:**

In total, 315 male and female children participated in the study and 11 participants were excluded, therefore, 304 participants completed the assessments. Anthropometric measurements were collected at the time of study, along with age, height, weight, circumference of the hand, hand span, hand length, palm length, and hand grip strength (HGS) was measured. Both decision tree and regression machine learning analyses were used to isolate the relative contribution of independent features in predicting the targeted grip strength of children.

**Results:**

Two predictive models were developed to understand the role of predictor variables in dominant hand HGS for both boys and girls. For boys, the decision tree was found to be the best model with the lowest error in predicting HGS. The respondents’ age, hand span, and weight were the most significant contributors to male hand grip strength. For the boys under 9.5 years of age, based on the decision tree analysis, weight (split at 27.5 kg) was found to be the most significant predictor. Furthermore, for the boys under 14.5 years of age, weight (split at 46.7 kg) remained the most important predictor. For boys 14.5 years and older, hand span was important in predicting handgrip strength. Backward regression was found to be the best model for predicting female hand grip strength. The *R*^2^ value for the model was 0.6646 and the significant variables were body mass index (BMI), hand length, hand span, and palm length, showing significance at a *p*-value of ≤0.05. This model predicted 66.46% of the variance in handgrip strength among the girls.

**Conclusion:**

Anthropometric factors played a significant role in hand grip strength. Age, weight, and a larger hand span were found to be significant in impacting male HGS, while BMI, hand length, and palm length contributed to higher grip strength among the girls.

## Background

Hand grip strength (HGS), referring to the ability to grasp and hold objects firmly, is a crucial aspect of motor skills development in children. It plays a significant role in numerous everyday activities, ranging from writing and drawing to participating in sports and carrying out various household chores. Often overlooked, grip strength deserves recognition for its impact on children's physical and cognitive development. While robust data are available for predicting grip strength in adults, very few studies have provided normative data among children and reference values for HGS are therefore lacking (1–3).

HGS has been found to be a significant predictor of physical fitness. Physical fitness has been found to influence academic performance and cognitive function, thus, it is important to measure HGS, especially among young children (4, 5). It is important to establish normative reference values for comparison to aid in future clinical management evaluations. Low levels of muscular strength are also associated with higher mortality rates in some individuals (6). A 20-year follow-up study found that low hand grip strength in early life predicts lower hand grip strength in adulthood, indicating a relationship with reduced muscular strength (7).

HGS in boys has been found to have an exponential progression in pubertal ages when compared to girls (1). Previous studies show lower HGS for the non-dominant hand for both genders (8). Along with gender and age, variables such as hand circumference, hand length, hand span, and forearm circumference are correlated to HGS (9, 10). A significant correlation has been identified between palm length and HGS. Gender has also been found to be significantly different in its relationship with HGS (11, 12).

Studies have proven that HGS among children varies according to gender, age, physical activity level, pubertal development, and body dimensions (13, 14). Muscle strength research in Saudi children is not so elaborate, and this can be explored to provide normative HGS reference data for preventive and follow-up measures (15). In addition, Saudi children's height and weight are lower when compared to their peers from Western countries, and hence, it is important to establish norms of grip strength specific to the country (16). There are studies that provide normative HGS data for Spain (17), Canada (16), Iran (8), and India (18) and only few in comparison for Saudi Arabia, which makes this research robust and necessary to assess and improve clinical management of HGS. This study aimed to develop predictive models to understand and isolate the feature significance of anthropometric and demographic variables in HGS of the dominant hand of young children (both boys and girls) in Abha, Saudi Arabia, using a Jamar dynamometer.

Assessing hand grip strength in young children holds significant value for several reasons. First, it serves as a gauge of their overall muscular strength and development. Second, it aids in detecting any muscle weaknesses or imbalances, which can then be targeted with specific exercises and therapies. Moreover, monitoring hand grip strength over time offers valuable insights into a child's growth and progress.

## Materials and methods

### Subjects

A total of 315 young children, both male and female, who were randomly selected from schools in the Aseer region, Abha, were evaluated. After employing simple random sampling, the children were assigned a unique identifier, and a computerized randomization tool was used to select the participants, ensuring that every eligible child had an equal opportunity to be included in the study. To ensure low selection bias, neither the researchers nor the participants were aware of the selection outcomes. This process was reviewed and received approval from the Ethical Committee at King Khalid University, ensuring transparency and adherence to ethical guidelines. In total, 11 participants were excluded because they did not complete all the evaluations and measurements, resulting in the enrollment of 304 young Saudi Arabian students aged 6–15 years in the study. After obtaining informed consent from their parents and/or guardians, they were provided with a detailed description of the study's purpose. The study included a total of 132 boys and 172 girls (56%) schoolchildren aged 6–15 years. A detailed age-based count of the children is provided in Table 1.

**Table 1:** Respondent count based on age category.

Age category (years)	Count
6	8
7	30
8	34
9	33
10	25
11	19
12	9
13	56
14	40
15	50

Children with a history of joint pain in the hands, fracture, comorbidities, infections, neurological disorders, or deformities related to upper extremities were excluded from the study. The sample was balanced by gender as a stratum. This was based on the World Bank Gender Data Portal, which indicates that “102.9% girls and 97% boys have completed secondary school in Saudi Arabia.” This shows that there are more girls than boys in Saudi Arabian schools. Hand dominance in the children was defined when the participating children expressed their preference for predominantly using one hand over the other in their daily activities. This study received approval from the Research Ethics Committee at King Khalid University (HAPO-06-B-001).

### Instrumentation and procedures

A Jamar hydraulic dynamometer (Hydraulic Hand Dynamometer, Fabrication Enterprises Inc., Irvington, NY, USA) was adjusted to the second handle position to measure HGS. The participants were allowed an initial practice with the device to ensure they were acquainted with the procedure and usage of the device.

### Measurement protocol

**Figure 1:**
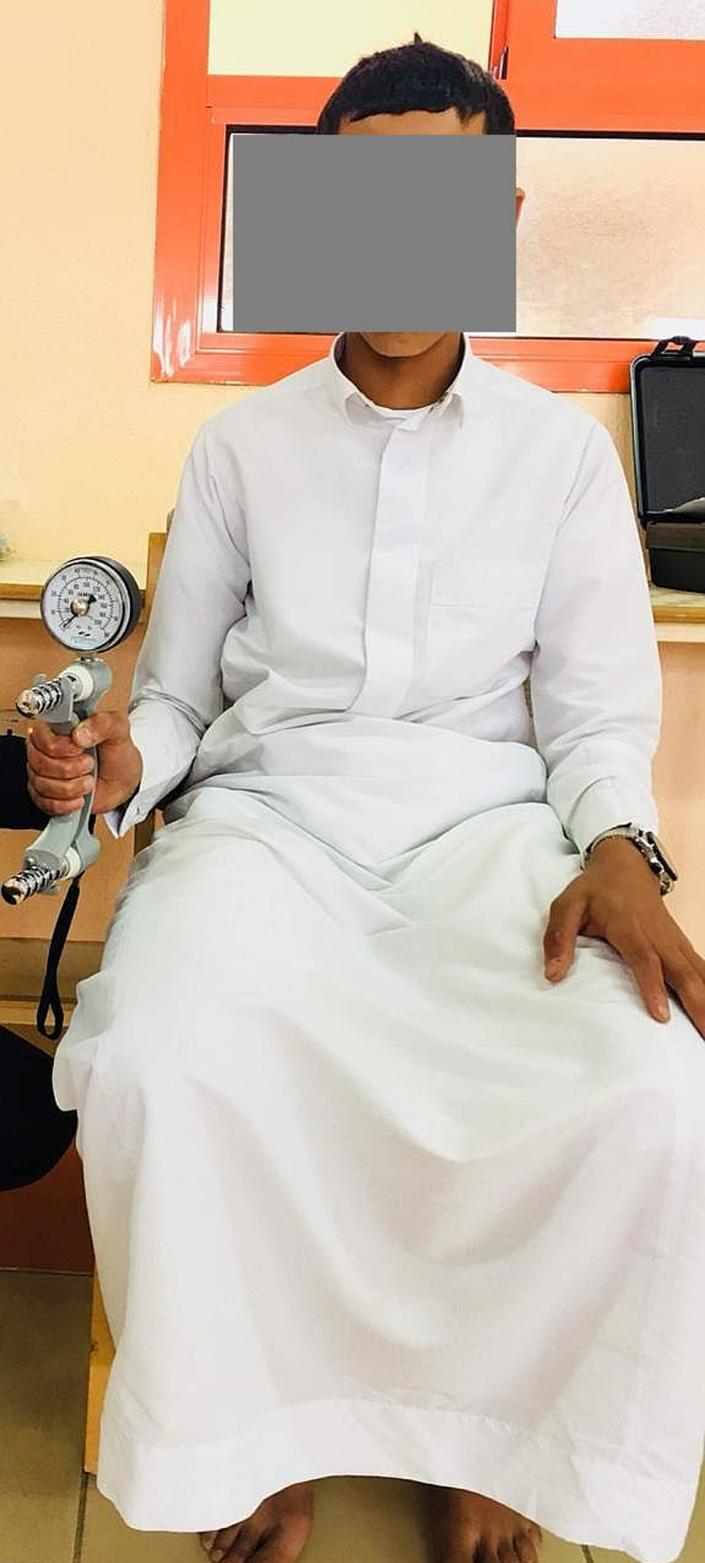
Measurement of HGS in sitting position.

To measure their HGS, the participants were comfortably seated in a chair with their back straight, ensuring the shoulder was in adduction with their elbow flexed at 90°, forearm in a neutral position, wrist positioned between 0°–30° of extension and 0°–15° of ulnar deviation, and feet flat on the floor (Figure 1) (19).

The examiner positioned the handheld dynamometer parallel to the long axis of their forearm, and the participants were instructed to squeeze the device as hard as possible for 3 s. The test was repeated three times, with a 3-min rest period between each trial. All measurements were recorded in kilograms and the maximum voluntary HGS was calculated as the mean value of the three trials for each participant.

### Anthropometric measurements

Anthropometric measurements, including age, height, weight, hand circumference, hand span, hand length, and palm length, were recorded at the time of the study.

All the participants were well-informed about the study procedures. Height was measured to the nearest 0.1 cm with a stadiometer (Pelstar, Alsip, IL, USA), while body weight was recorded to the nearest 0.1 kg using a portable digital weighing scale (Camry, model EB 932, China, Ltd.). When measuring anthropometric variables other than height and weight, the participants were instructed to sit comfortably. Their body mass index (BMI) was calculated using the following formula: body mass (kg) divided by standing height squared (m^2^). Hand dimensions were measured based on the National Aeronautics and Space Administration (NASA)-1024 guidelines (20), with hand circumference measured along the hand arch to the highest level of the palm (21). For the hand span measurement, the distance from the tip of the thumb to the tip of the little finger was measured after the participants were instructed to fully extend and spread their hand on a piece of paper placed on the table. For the hand length measurement, the distance from the tip of the middle finger to the midline of the distal wrist crease was measured, with the hand and forearm positioned in supination on a table. Palm length was measured from the base of the middle finger to the base of the palm using an inch tape.

### Predictive analysis using machine learning

Predictive analytics using machine learning techniques are different from statistical methods. Machine learning models provide the most accurate predictions, while determining conclusions about the correlations between variables is the aim of statistical models. In this study, predictive analytics models were developed with anthropometric variables as the independent features and grip strength as the dependent variable.

Two software programs were used in the study. SAS Guide 8.4 was used for data analysis (such as descriptive analysis and correlations), while machine learning analysis was performed using SAS Enterprise Miner 15.3. Miner is an interactive data mining tool that uses process flow diagrams to map the mining process. It provides a guided user interface in a drag-and-drop tool for creating a mining workflow. We developed a predictive analysis model with HGS as the target variable and palm length, hand span, and hand circumference as the predictors.

Predictive analysis is a mathematical process used to find solutions by extracting information from historical data sets, identifying patterns and trends and predicting future outcomes. One differentiating pattern of predictive analysis compared to data analysis is the use of train and test samples. This allows us to make future predictions based on the test sample and ensure that the data results are not overfitted or underfitted, thereby minimizing errors. Whilst data analysis can also make predictions, predictive analysis models can provide confident predictions by using techniques specialized for optimized decision-making.

A 70:30 train test data partition is used to ensure cross-validation in machine learning, with errors calculated based on average squares. Such partitioning can help in making accurate estimations of the prediction quality for future data. After the model is trained, the test model helps in making predictions against the trained model, minimizing discrepancies in future data predictions.

Two predictive analyses, decision tree and linear regression, were conducted. Decision tree algorithms create a visual flowchart with branches to display the outcomes of the variables. This flowchart model (with a tree-like appearance) uses “conditional algorithms” to classify data with branches showing different possible outcomes, making it excellent at interpreting data. The goal of the decision tree was to create a training model to predict the value of the target variable, i.e., HGS. Regression analysis helps in investigating the relationship between the target and independent variables (features). Multiple regression models—full, forward, backward, and stepwise—were run to provide a comparison to the decision tree predictions. In a predictive analysis environment, multiple predictive tools are compared to identify the correct metrics for optimized decisions with the lowest prediction errors.

A model comparison node is then used to compare these different analytics techniques. This comparison is based on the lowest error rates and the significant variables identified by the technique are used to create a data narrative and determine significant predictors.

### Data analysis and predictive analysis

Data are expressed as means, medians, standard deviations, and ranges, using SAS Enterprise Guide data analysis software. The skewness values showed that the data were well within the normal distribution of ±3 SD. Correlation analysis, using both Pearson's correlation and point biserial correlation, was used to evaluate the relationship between HGS and other variables.

Two predictive analyses, decision tree and linear regression, were conducted using SAS Enterprise Miner software. The model comparison showed that the decision tree showed the lowest average square error (Figure 3) for predicting male hand grip strength, while regression was the best model for predicting female hand grip strength. Statistical significance was set at *p* < 0.05 for both the correlation and regression analyses.

## Results

In total, 315 participants were evaluated. However, 11 participants were excluded as they did not complete all the measurements. Therefore, 304 participants completed the assessments and enrolled in the study.

Table 2 summarizes the demographic and anthropometric characteristics. The mean age was 10.81 ± 2.79 (median also showing age 10), and the mean weight, height, and BMI were 39.95 ± 15.68 kg, 139.92 ± 17.84 cm, and 19.80 ± 4.511 kg/m^2^, respectively. A side-by-side description of the median values and the skewness is also provided in Table 2. There were no significant differences in hand length, palm length, hand circumference, hand span, and HGS between the dominant and non-dominant hands of the participants. All the respondents reported being right hand dominant.

**Table 2:** Mean and SD of the demographic and anthropometric characteristics of the participants.

Variable	Missing data	Minimum	Maximum	Mean	Median	Standard deviation	Skewness
Dominant hand HGS	0	3.26	64.27	23.03	17.96	15.51	0.94
Weight	0	16.9	85.8	39.95	37.2	15.68	0.71
Age	0	6	15	10.81	10	2.79	0.142
BMI	0	12.60	45.8	19.80	19.1	4.51	1.09
Hand Length	0	11.2	23	16.07	16.2	2.15	0.23
Palm Length	0	6.72	13.8	9.64	9.72	1.29	0.24
Hand span	0	13.2	23.6	18.31	18	2.37	0.24
Height	0	105	175	139.92	139.6	17.84	−0.03
Hand circumference	0	14	23.8	17.81	17.4	2.25	0.58

Mean and standard deviation of hand length, palm length, hand circumference, hand span, and hand grip strength of the dominant hand.

A correlation analysis of all the features of the dominant hand is shown. Table 3 demonstrates the correlations between the variables and HGS. A high correlation between dominant hand palm length, hand span, and hand circumference and HGS was found (22). A significant negative correlation was found between being female and HGS.

**Table 3:** Correlation matrix of hand grip strength of the dominant hand with demographic and anthropometric variables.

Variable	Handgrip strength
Age	0.684
BMI	0.348
Weight	0.615
Height	0.633
Male	0.824
Female	−0.824
Hand circumference	0.755
Hand length	0.621
Hand span	0.695
Palm length	0.622

Correlations between dominant hand palm length, hand span, hand circumference, and demographic variables and hand grip strength.

### Predictive modeling for HGS

**Figure 2:**
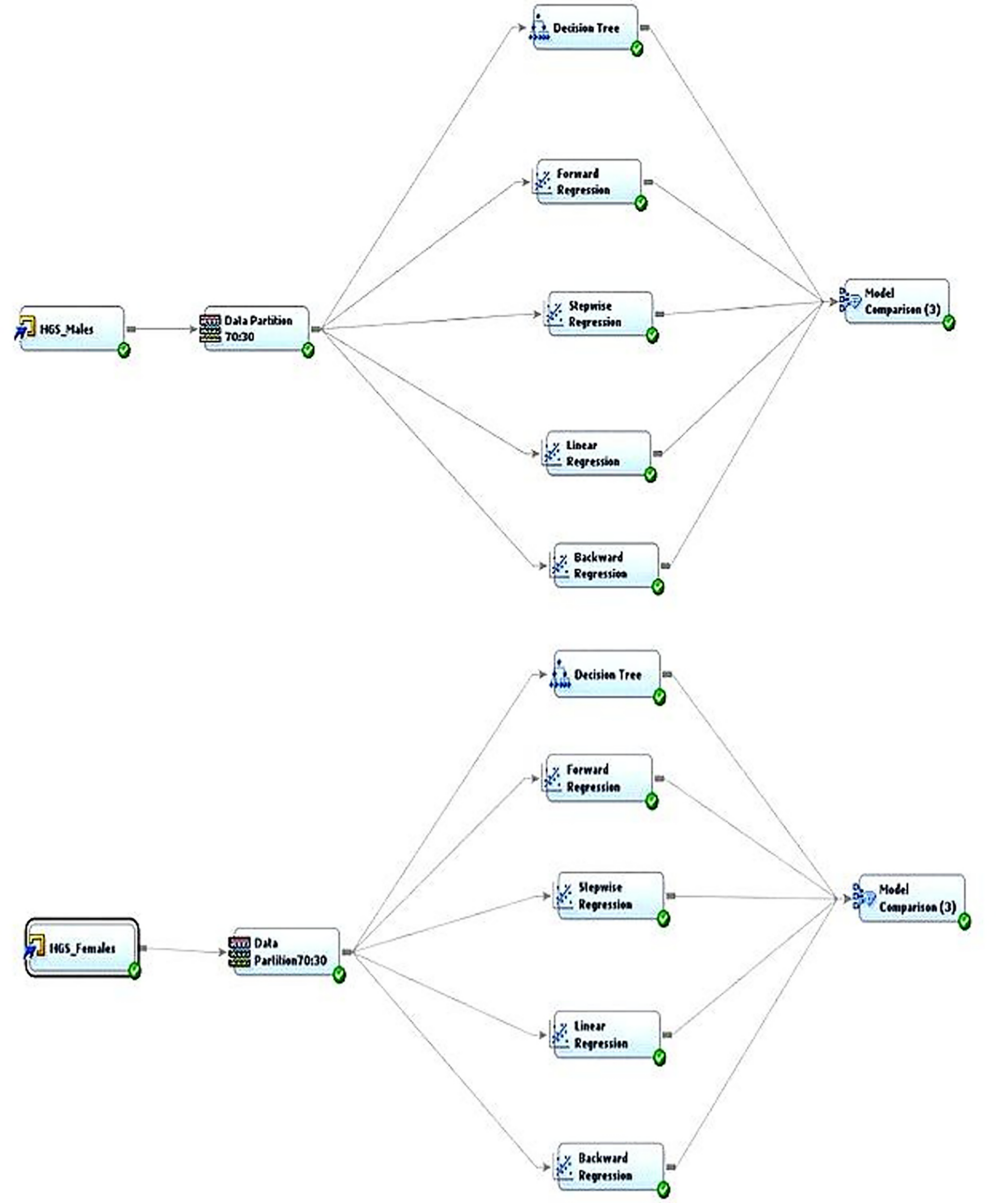
Outlines of the two predictive analytic model diagrams developed for measuring HGS for both boys and girls.

Two predictive models were developed to understand the role of predictor variables in dominant hand HGS in both boys and girls. Figure 2 outlines the two predictive analytics model diagrams developed for measuring HGS in both boys and girls. The data were portioned as 70:30 train test data. In machine learning, the “train test validation” requirement involves dividing a dataset into three separate portions: a training set used for training the model, a validation set used to fine-tune hyperparameters and assess model performance during training, and a test set used to evaluate the final model's performance on entirely unseen data, ensuring an unbiased assessment of its generalization capability. Two types of predictive analyses, decision trees and regression analysis (full, forward, backward, and stepwise), were run and the best prediction model was chosen based on the lowest average square error. Backward regression was found to be the best prediction model for understanding HGS in girls and the decision tree [average square error (ASE) = 44.3] was found to be the best predictive model for boys in comparison to the regression tree (full regression ASE = 44.3, forward regression ASE = 44.5, and stepwise regression ASE = 44.6). Backward regression was found to be the best predictive model for females, in comparison with other regression models and decision trees (backward regression ASE = 8.97; full, forward, and stepwise regression ASE = 9.32; and decision tree ASE = 9.42). Table 4 outlines the comparisons of the predictive models and models with the lowest errors were selected because of their better prediction power.

**Table 4:** Comparison of the predictive models.

Gender	Target	Model	Branches/variables	Lowest validation average square error
Male	HGS	Decision tree	2	44.3
Female		Backward regression		8.97

Average squared values for a comparison of the predictive models.

#### Male hand grip strength

**Figure 3:**
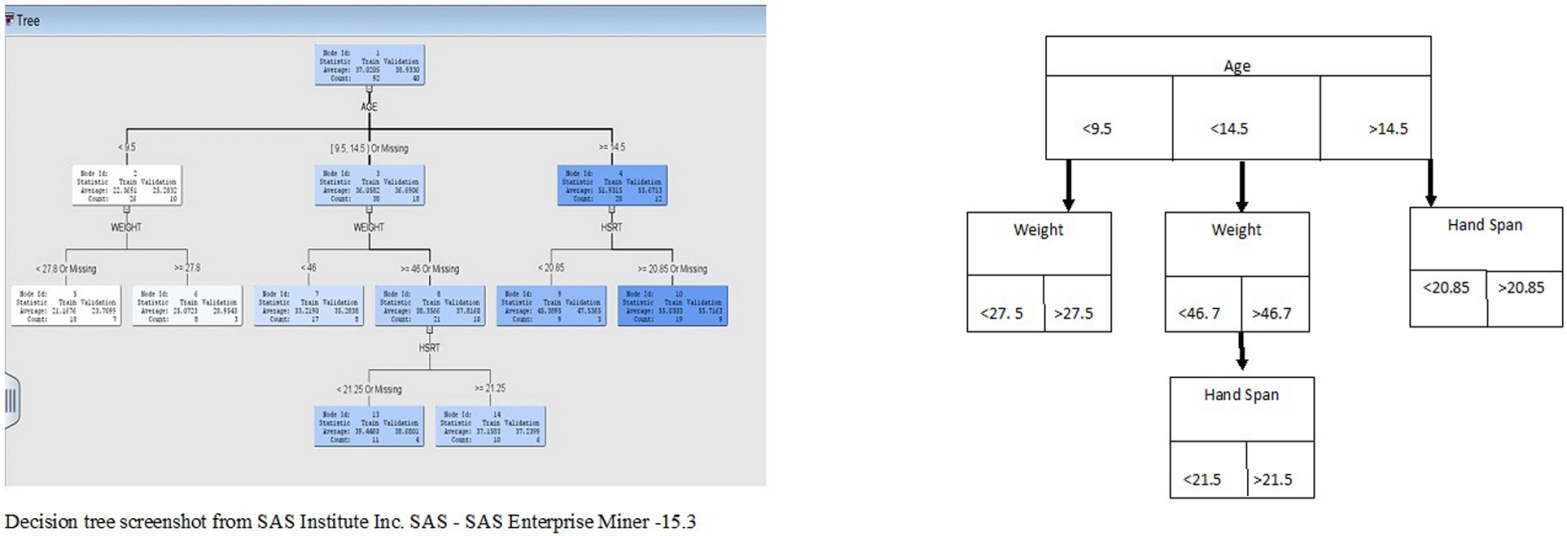
Male HGS decision tree branching.

A predictive decision tree model was found to be better than regression to predict HGS in boys. It was found that age, hand span, and weight were the most significant contributors to male HGS, based on the first leaf split. For boys younger than 9.5 years of age, weight (split at 27.5 kg) was found to be the most significant predictor based on the logworth split. Logworth is calculated as −log_10_(*p*-value), where the *p*-value is determined by considering the number of different ways splits can occur on decision trees. Thus, for boys younger than 9.5 years of age, weight was the most significant variable that predicted HGS if they weighed above or below 27.5 kg. Furthermore, for boys younger than 14.5 years old, weight (split at 46.7 kg) remained the most important predictor. However, if they weighed more than 46.7 kg, then hand span (greater than 21.5 cm) was the most important predictor. For boys older than 14.5 years, hand span was an important variable for predicting grip strength. Figure 3 illustrates the decision tree with all its branches.

#### Female hand grip strength

Backward regression was found to be the best model for predicting female HGS, compared to other methods including full, forward, and stepwise regression models. Backward regression, also known as backward elimination, is a stepwise regression approach used to build a statistical model by removing predictors sequentially. The *R*^2^ value for the model was 0.6646, with the significant variables being BMI, hand length, hand span, and palm length. This means that 66.46% of the variance in HGS can be explained by body mass, hand length, and palm length. Machine learning analysis generally does not use *p*-values because there is usually no reasonable hypothesis about the underlying distribution, so significance values do not apply, but variable significance is still shown based on the *p* < 0.05 criteria. Table 5 outlines the significant predictors of female hand grip strength.

**Table 5:** Regression model for predicting hand grip strength in girls.

Parameter	*p*-value
BMI	0.031
Hand length	0.025
Hand span	0.039
Palm length	0.032
*p-*value (≤0.05 significance level)	

Significant predictors of hand grip strength in girls *p*-value (≤0.05 significance level).

## Discussion

The main purpose of this study was to identify the impact of gender, hand circumference, hand span, palm length, and BMI on HGS among children aged 6–15 years in Saudi Arabia. Machine learning predictive analysis was applied to identify correlated variables as potential predictors and to facilitate the interpretation and visualization of HGS data. Since there was a notable finding in the correlation analysis, where being female had a negative correlation with HGS, two predictive models were implemented to understand how gender differences influence the predictive variables for HGS.

The anthropometric factors studied were found to have significant positive correlations with HGS. The features in the present study have been researched rigorously, and the correlation between the various anthropometric factors, including age, weight, and BMI, and HGS has been previously established (1, 23, 24). In general, the findings of the present study agree with previous studies performed in similar populations. Grip strength was found to be related to the anthropometric determinants and hand dimensions researched in this study.

A Chinese study using decision tree predictions found a significant difference in the hand grip strength of women. While this study aimed to find predictors of handgrip strength in diabetes mellitus, they found that women showed significantly different HGS compared to men (25). Another study investigating cardiorespiratory illness based on HGS also found that female HGS was relatively lower than male HGS (26). While the effect of gender on HGS has been extensively researched, most research studies have focused on an aging population and HGS as an indicator of frailty (27, 28).

The impact of culture and dietary patterns have been found to be a reason for increased grip strength in boys (29). Grip strength is a crucial indicator of overall fitness and can reflect various health aspects in the 5–19 age group. Furthermore, multiple studies have examined the position during HGS testing as a predictor of changes in grip strength (30, 31). A lack of physical activity and insufficient vitamin D have also been found to have significant effects on HGS (32, 33) A study showed that grip strength varies significantly among different societies, for instance, in populations in the United States, the United Kingdom, Turkey, Iran, Switzerland, and Australia, and a general database of Western countries demonstrate higher average grip strength across all age groups and both sexes compared to the Saudi population (34).

The results of this study could be specifically relevant to a place such as Abha, Saudi Arabia, given its altitude and cultural indicators. The result of this study has major implications in understanding the necessity for adequate children's nutrition and the requirement to provide balanced diets to young children. A recent study in 2021 found that 69% of Saudi children did not meet the recommended daily intake of fruits and vegetables (35).

Previous studies have indicated a positive association between the strength of grip and the ages of boys, especially between 6 and 12 years old, and that the HGS of boys is stronger than that of girls (29, 33). Dominant handgrip has been associated with hand length (36), palm length, hand span, and other anthropometric characteristics (37, 38) in previous studies. The result of this study shows that for healthy boys, only age, weight, and hand span were important variables in predicting HGS. This could be a significant reason why some previous studies did not find significant differences in HGS between genders (39, 40). However, the result of this study is also consistent with previous research that found that boys are much stronger than girls, particularly after 11 years of age (41).

The results provide a better understanding of how gender differences can lead to changes in HGS. For example, for girls, it is determined by BMI, hand length, hand span, and palm length. BMI was a significant factor when predicting female HGS, and this is an indicator of how body size differences in boys and girls can influence grip strength. This can also be related to previous research that showed a lack of physical activity led to reduced HGS in women (32). Another study determined that hand size must be included when assessing hand grip strength in women (42). Significant correlations between forearm length, hand circumference, and hand measurements, and grip strength have been found among older adults (43). Studies have also shown that female HGS does not change significantly after 13 years of age (44) and the result of our study indicates the necessity for more physical activity for girls in the study area.

The result of this research provides a detailed, self-explanatory, and visually simple understanding of the grip strength of children. Such an in-depth analysis of these predictor features will aid in making accurate estimations of hand-based parameters.

The present study has some limitations, as it did not compare dominant and non-dominant hand grip strength. Furthermore, the sample size may have been insufficient to detect smaller differences in grip strength, potentially affecting the statistical power of the study. Moreover, the study did not account for socioeconomic factors, which might influence both grip strength and overall health. Data on the nutritional status and level of exercise of the children who participated in the research study can be added in the future to provide additional research insights.

## Conclusions

Handgrip strength in children aged 6–15 years is mainly affected by factors including gender, age, hand span, hand length, and hand circumference. These factors follow a specific order of importance based on grip strength measurements. For boys, a larger hand span, age, and weight significantly contributed to higher grip strength. In girls, the key variables included BMI, hand length, hand span, and palm length. Monitoring handgrip strength over time can offer valuable insights into a child's growth and development.

## Data Availability

The raw data supporting the conclusions of this article will be made available by the authors, without undue reservation.
